# Genetic analysis of suicide: a sample study in Tuscany (Central Italy)

**DOI:** 10.1080/20961790.2020.1835156

**Published:** 2022-05-30

**Authors:** Martina Focardi, Barbara Gualco, Vilma Pinchi, Norelli Gian-Aristide, Regina Rensi, Elisabetta Pelo, Ilaria Carboni, Ugo Ricci

**Affiliations:** Division of Genetic Diagnostics, University Hospital “Careggi”, Florence, Italy

**Keywords:** Forensic sciences, forensic genetics, suicide, *MAOA*, *HTR2A*, *SCN8A*, *NOS3*

## Abstract

Many studies have examined the genetic contribution to suicide. However, data on suicide in the Italian population are scarce. We therefore aimed to address this gap by investigating a cohort of 111 Italians for whom a verdict of suicide had been declared in court in Florence, Italy between 2007 and 2017. This cohort included 86 men and 25 women. DNA samples were obtained from tissues or blood, and 22 genes from multiple neurobiological pathways previously shown to be associated with the pathogenesis of suicide were analysed. Next-generation sequencing was used to compare these gene sequences with those from a large, normal population. In this study, we identified 19 gene variants that were present at significantly lower frequencies in our Italian cohort than in the general population. In addition, four missense mutations were identified in four different genes: Monoamine Oxidase A (*MAOA*), 5-Hydroxytryptamine Receptor 2 A (*HTR2A)*, Sodium Voltage-Gated Channel Alpha Subunit 8 (*SCN8A*), and Nitric Oxide Synthase 3 (*NOS3*). Our study identified several potential genetic links with suicide in a cohort of Italians and supports a relationship between specific genetic variants and suicidal behaviour in this population.

## Key points

This study shows a genetic analysis of suicide.

This study examines a cohort of 111 Italians for whom a verdict of suicide had been declared in court in Florence, Italy between 2007 and 2017. DNA samples were obtained from tissues or blood, and 22 genes from multiple neurobiological pathways were investigated.

The study supports a relationship between specific genetic variants and suicidal behaviour.

## Introduction

Over 700 000 people take their own life every year throughout the world, and many more attempt suicide. Each suicide is a tragedy that affects families, communities, and entire countries and can have long-lasting effects on the people left behind. Suicide can occur throughout the lifespan, but was the second leading cause of death among 15–29-year-olds globally in 2016 [1].

According to the latest available data at the Italian National Institute of Statistics (ISTAT), the 2015 suicide rate in Italy was 8.4 per 100 000 inhabitants [2]. This is significantly lower than the world average and well below the European average, which is currently 15.4 suicides per 100 000 inhabitants. Among young Italians between the ages of 14 and 24, the suicide rate is slightly higher than the European average at 8.5 per 100 000 inhabitants. For this age group, suicide represents the second most common cause of death after road accidents. Over 77% of suicide cases in Italy involve male victims. This rate is three times higher than in females. The most common methods of suicide are hanging, self-poisoning with a pesticide, and firearms [3]. According to the 2017 ISTAT data [2], the most common methods of suicide were hanging or suffocation (48.9%), jumping (19.2%), and firearms (11.3%).

Backed by scientific evidence, we now know that there is a genetic predisposition to manifesting suicidal behaviour [4–8]. The earliest studies searching for genetic susceptibility loci in suicidal behaviour used candidate gene approaches. However, when the studies focusing on specific genetic systems failed to produce consistent results, multiple large-scale studies were done to identify genetic contributors to suicidal behaviour. Emerging diagnostic applications are frequently used in medical genetics, medical subspecialties such as neurogenetics [9], and suicidality outcomes [10]. Generally, the samples/cohorts used for these investigations were not initially designed to study suicidal outcomes but for few exceptions [11, 12]. However, the mechanism and magnitude of genetic contributions to suicidal behaviour are still poorly understood.

Decreased serotonergic transmission is one of the most consistent findings in genetic studies of suicide [13]. However, some studies have reported contradictory results, suggesting that the genetic contribution to suicidal behaviour is quite complex.

The dopamine receptor D4 (*DRD4*) gene is situated in a chromosomal region containing CpG islands that host a number of imprinted genes. Variable number of tandem repeats (VNTRs) in the third exon of this gene have been investigated by several groups [14–16], as well as single nucleotide polymorphisms (*SNPs*) reported in certain studies [17].

Many SNPs, VNTRs, and alleles in 5-hydroxytryptamine receptor (*HTR1A*, *HTR1B, HTR2A,* and *HTR2C)*, as well as in serotonin transporter (*SLC6A4*) genes, can influence aggressive or suicidal tendencies [18, 19]. Therefore, we included these polymorphisms in the current study. Moreover, the serotonin-transporter-linked polymorphic region (*5-HTTLPR*) is reportedly a predictor of short-term risk of suicide reattempts [20]. Variations in the Adrenoceptor Alpha 2 A (*ADRA2A*) gene linked to the average population [21, 22] are described in cases of suicide and withdrawn behaviour. It has been established that the neuronal isoform of nitric oxide synthase (*NOS I*) modifies diverse behaviours, which include aggression [23, 24]. We examined the SNP variation in our population and control group to verify any statistically relevant difference rather than analyse the promoter VNTR in *NOS I* Exon 1f. Effects of polymorphisms in the endothelial nitric oxide synthase (*NOS III*) gene on suicide [25–27] were also investigated. The importance of Tyrosine 3-monooxygenase/tryptophan 5 monooxygenase activation protein epsilon (*YWHAE*) gene polymorphisms (*rs1532976*, *rs3752826*, and *rs9393*) in suicidal behaviour was studied in Russian and Tatar ethnic groups in the Republic of Bashkortostan [28, 29].

Studies examining the link between monoamine oxidase *A (MAOA)* VNTRs [30, 31] show a statistically significant tie to psychiatric patients in certain cases. The corticotropin-releasing hormone receptor 2 (*CRHR2*) gene and suicidality in bipolar disorder have previously been studied [32]. Other cases found the tryptophan hydroxylase 1 (*TPH1*) and *TPH2* genes [33, 34] to be related, but there were contradictory results. The catechol-O-methyltransferase (*COMT*) gene variation, especially the Val108/158Met polymorphism, has recently been linked to suicides [35].

Genetic variation can also affect factors that increase suicide risk. Specific alleles or SNPs in the brain-derived neurotrophic factor (*BDNF*) gene have possible neurobiological impacts on the etiology of schizophrenia [5]. Selected SNPs in the dopamine receptor (*DRD2*) gene [36] are possible genetic risk factors for the development of alcohol addiction and other associated disorders. These SNPs have been studied in attempted suicides [37]. A review by Mandelli and Serretti [38] reported evidence that supports both inheritable and environmental influences in depression and suicidal behaviour risk for the angiotensin-converting enzyme (*ACE*). Gene-variants in or adjacent to the sodium channel voltage gated type VIII alpha polypeptide (*SCN8A*), vesicle-associated membrane protein 4 (*VAMP4*), and prenylated Rab acceptor 1 (*RABAC1*) genes are over-activated in attempted suicides [38]. Finally, significant ties were found with variations in rs6740584 (*CREB1*) polymorphisms in cAMP-response element binding proteins 1 [39].

In this study, we present research performed at the forensic medicine laboratory in Florence (central Italy) aiming to further understand the relationship between specific genes and suicide. A large panel of 22 genes was generated to examine if significant statistical differences in alleles frequencies were present populations. These genes included *DRD4*, *HTR1A*, *HTR1B*, *HTR2A*, *HTR2C*, *5-HTTLPR*, *ADRA2A*, *NOS I*, *NOS III*, *YWHAE* (14-3-3ε gene), *MAOA*, *CRHR2*, *TPH1*, *TPH2*, *COMT*, *BDNF*, *DRD2*, *ACE*, *SCN8A*, *VAMP4*, *RABAC1*, and *CREB1*. These genes were chosen through a literature search of previous articles associated with psychiatric disorders, suicide groups, and attempted suicides.

## Materials and methods

### Cohort history

After obtaining a careful bibliographical history of the subject, we performed an anamnestic-circumstantial investigation. The variables considered were sex, age, methods of suicide, and motives for suicide. The data were then compared with ISTAT reports from 2015 for “deaths and causes of death” and “suicides and suicide attempts” [2]. Both categories were necessary to verify agreement with the national database. This information was also important when comparing subjects who had previously attempted suicide or had psychiatric disorders. We also identified the gene polymorphisms.

### Samples

A total of 111 samples (blood or tissue) were collected from a cohort of 111 suicidal subjects (86 males and 25 females) with an average age of 40.18 ± 14.80 years, which were available at the Institute of Forensic Medicine, University of Florence, Italy. Prior to the start of the study, ethical approval was obtained by the Regional Ethics Committee for Clinical Trials in Tuscany (section Central Area, Register Number Bio. 16. 003). All data were approved for use in this study from Etic Committee of Department of Helath Sciences, University of Florence, Italy.

### DNA isolation

Genomic DNA was isolated from blood samples using a QIAsymphony DSP DNA Kit and a QIAsymphony instrument (Qiagen, Hilden, Germany) following the manufacturer's protocol. DNA from tissues was extracted using a QIAmp DNA Tissue Kit and a BioRobot EZ1 Advanced Workstation (Qiagen). The concentration of the isolated DNA was determined using a Qubit dsDNA Broad Range Assay Kit (Life Technologies, Carlsbad, CA, USA).

### Next-generation sequencing (NGS)

Targeted sequencing of 22 candidate genes was performed using the Agilent HaloPlex target enrichment assay (Agilent Technologies, Santa Clara, CA, USA) according to the manufacturer’s protocol. The samples were sequenced on an Illumina MiSeq platform (Illumina, San Diego, CA, USA). Regions of the selected target genes (ROI, region of interest) covered by less than 20 reads were defined as “low coverage regions”. Although some polymorphisms may have been identified in these regions, the presence of additional polymorphisms cannot be excluded. Sequencing probes for targeted regions were designed using the SureDesign Custom Design Tool software (Agilent Technologies). The following genes were analysed: *DRD4*, *HTR1A*, *HTR1B*, *HTR2A*, *HTR2C*, *5-HTTLPR*, *ADRA2A*, *NOS I*, *NOS III*, *YWHAE*, *MAOA*, *CRHR2*, *TPH1*, *TPH2*, *COMT*, *BDNF*, *DRD2*, *ACE*, *SCN8A*, *VAMP4*, *RABAC1*, and *CREB1*. Using the SureDesign tool, the probes were targeted to a total of 211 regions, including 2 810 amplicons with a theoretical target coverage of 99.87%. The obtained region size was found to be 47.921 kilobases. For each gene, both coding and non-coding (regulatory) regions were included in the sequencing. The coding regions contained exons including all alternative transcripts as defined by Ensembl, GENCODE, RefSeq, or “UCSC Genes” of the UCSC Genome Browser [40].

### Genetic analysis

Read quality was verified with FastQC, and reads were aligned to the reference human genome hg19 with BWA MEM (Burrow-Wheeler Aligner) (http://www.bioinformatics.babraham.ac.uk/projects/fastqc). Genome Analysis Toolkit (GATK) version 3.3 (https://gatk.broadinstitute.org/hc/en-us) was used to recalibrate base qualities and realign reads around insertions or deletions (InDels). Single Nucleotide Variants (SNVs) and InDels were identified by the Unified Genotyper module of GATK. Genomic and functional annotations of detected variants were made by Annotate Variation Software (ANNOVAR, version 14 December 2015) (https://annovar.openbioinformatics.org/en/latest/). Detected variants were distilled on the basis of their exonic function, allele frequency, and presence in variants databases, such as the current release of dbSNP [41], 1000 Genomes Project (http://ftp.ncbi.nih.gov/), Exome Sequencing Project (http://evs.gs.washington.edu/EVS/), gnomAD (The Genome Aggregation Database), and several prediction scores (Sorts Intolerant From Tolerant (SIFT), MutationTaster, Polymorphism Phenotyping v2 (PolyPhen-2), LRT, CADD, Gerp, and PhyloP). To identify clinically relevant variants, SNVs and InDels were filtered based on frequency, function (coding, 5′ or 3′ junctions), evolutionary conservation of the affected nucleotide, and pathogenic potential by *in silico* predictive algorithms. Coverage statistical analysis was performed using the Depth Of Coverage tool in GATK. BASH and R custom scripts were used to obtain the list of low coverage (≤20X) regions per sample. Confidence of identified variants was determined according to the Best Practice Guideline of GATK regarding hard-filtering recommendations for selected germline short variants. Hard-filtering involves choosing specific thresholds for one or more annotations and removing any variants with annotation values above or below the set thresholds. Based on the hard-filtering recommendation, the variants that did not meet the following criteria were filtered out: MappingQualityZero (MQ0) ≥ 4 & (MQ0/(1.0* QualByDepth (DP)) > 0.1) | Quality (QUAL) <10. In particular, SNVs with QualByDepth (QD) <2.0 ǁ FisherStrand (FS) >60.0 ǁ RMSMappingQuality (MQ) <40.0 ǁ HaplotypeScore > 13.0 ǁ MappingQualityRankSumTest (MQRankSum) <−12.5 ǁ ReadPosRankSum<−8.0 were excluded. For InDels, variants with QD < 2.0 ǁ FS> 200.0 ǁ ReadPosRankSum<−20.0 were filtered out. None of candidate variants identified were excluded based on pathogenicity prediction obtained using *in silico* tools.

### Statistical analysis

The ExAC_ALL application (https://bredagenetics.com/il-database-exac-una-delle-risorde-piu-utili-per-la-comunita-scientifica/?lang=it) was used for statistical analysis of the data. For each DNA variant frequency in the general population, the number of alleles and total number of alleles in the database were detected. The Fisher test was performed to verify if a statistically significant variation was present between our dataset and the general population. The *P*-values of the single- and two-tailed Fisher test were corrected for using the Bonferroni test and the Benjamini and Hochberg tests. The significant variants of the correct Fisher test are displayed in the alignment file (bam file), using the IGV (Integrative Genomic Viewer) display browser, to evaluate if these variants could be misleading. For this purpose, all the significant variants present in at least two samples were visualised in IGV, excluding synonymous and intronic variants; in addition, some variants present in single samples and synonymous variants were investigated.

## Results

### Anamnestic data

To begin to understand the genetics of suicide in the Italian population, we collected data from 111 Italians who had committed suicide between 2007 and 2017. Table 1 shows some anamnestic results. First, the frequency of committing suicide is far greater in males (77.0%) than in females (23.0%) in Italy. The data suggest suicide follows an irregular course and is much higher in defined age groups. At the time of death, most victims were between 25 and 35 years old, with a higher percentage under 30 and between 40 and 64 years old.

**Table 1.** Anamnestic results (percentage values), *N* = 111.

Items	Percentage values (%)
Sex	
Male	77.0
Female	23.0
Methods of suicide	
Hanging	32.0
Fire weapon	20.0
Jumping to one’s death	18.0
Suffocation by plastic bag	8.5
Collision	8.5
Stabbing	8.0
Ingestion of drugs	5.0
Motives of suicide	
Psychiatric disorders	32.3
Drug treatment*	23.0
Previous attempts or suicidal ideation	13.1
Work or economic problems**	10.9
Grief caused by separation/divorce	6.9
Suicide in prison/psychiatric hospital	6.3
Under influence of alcohol/drugs	4.3
Homicide/suicide	3.2

* Suicide that occurred while the patient was undergoing drug therapy (tranquilisers, anxiolytics, antipsychotics) for psychological or psychiatric problems.

** Loss of work, bankruptcy, difficulty in supporting oneself, or general sense of social unease.

For the modalities of suicide (Table 1), the primary method was hanging (32%), followed by firearms (20%), jumping (18%), collision (8.5%), suffocation (8.5%), stabbing/cutting oneself (8%), and drug ingestion (5%). Motives and contributing factors were obtained from the police reports with family members and others close to the victims. Overall, the main motive of suicide in these cases was psychiatric disorders (32.3%).

### Genetic survey

The Fisher test, corrected for false discovery rate (FDR), identified 19 variants in our series with a different, statistically significant frequency compared with that of the ExAC_ALL analysis (*P*-value adjusted > 0.05) (Table 2). Of the gene variants we identified, seven had never been previously observed, seven had a lower frequency than the control population, and three had a frequency of less than 0.5% compared with the general population.

The seven variants observed in our series that had never been reported in the ExAC_ALL database were identified in single samples, none of which had related psychiatric precedence and none were linked to suicide. There were six exonic variants (*SLC6A4*, *CREB1*, *MAOA*, *HTR2A*, *SCN8A*, *and NOS3*) and one in the 5′ untranslated region (5′ UTR) of the *DRD2* gene. Two of the six identified exon variants are synonymous mutations (do not change the encoded amino acid), one in *SLC6A4* and one in *CREB1*, while four are missense mutations (*MAOA*, *HTR2A*, *SCN8A*, *and NOS3*). The variant in the *MAOA* gene, on the X chromosome, was identified as hemizygous in a male subject and is present in exon 8 (NM_001270458: c.286C > T; p.R96W) with an amino acid change from arginine to tryptophan. The *SCN8A* variant (NM_001177984: c.1336G > T; p.A446S), located in exon 10, shows an amino acid change from alanine to serine. The *NOS3* variant (NM_001160109: c.624C > A; p.F208L) shows an amino acid change from phenylalanine to leucine. The *HTR2A* variant (NM_000621: c.1334A>G; p.M445T) shows an amino acid change from methionine to threonine. These non synonymous SNVs changed the protein product of these genes and their biophysical properties, suggesting that the function of the protein might also be altered. The effects of non synonymous changes on the structure and function of a human protein were predicted using SIFT, PolyPhen-2, and MutationTaster. Table 2 shows the results obtained for these four genetic variants. Although the SNV observed seven base pairs upstream of the *DRD2* transcriptional start site would not influence the protein sequence, it could possibly have an impact on the transcriptional activity of the host or distal genes by affecting the binding affinity of transcription factors or microRNAs.

The seven variants with lower frequency observed in our series compared with those of the general population showed a frequency > 14% in the ExAC_ALL analysis. Two of these SNPs were located in the coding region of the *DRD4* gene: one was a synonymous variant and the other was a non-frameshift deletion. The synonymous variant in *DRD4* (rs762502, *P* = 2.1E–23) is present in the third exon and is a VNTR previously shown to be associated with suicidal behaviour [19]. A frameshift deletion of 12 bases (del GCGGGGGCATCT) is located in exon 1 of the *DRD4* gene (rs572586776, *P* = 1.74E–5). In our series, it was identified in only two patients with an overall frequency of 1% compared with 15% in the general population. These two subjects were heterozygous for this variant and had no psychiatric precedent, nor were any suicide attempts reported. Four variants with a potential protective role were present in the *ACE* gene. Of these, one is intronic and three are synonymous mutations. Their frequency in the general population is about 50%, while in our series, the frequency varied between 30% and 40%. Finally, the variant in the *NOS3* gene is a missense mutation in exon 7 that leads to an amino acid change of aspartic acid to glutamic acid (NM_001160109: c.894T > G; p.D298E, rs1799983). Its frequency varies from 75% in the general population to 65% in our cohort (*P* = 0.00816) and is reported as “benign” in the ClinVar database.

**Table 2.** Detailed list of identified gene variants.

chr	start	end	Ref	alt	Function	Gene	GeneDetail.refGene	ExAC_ALL_allele_freq
chrX	43590527	43590527	C	T	Exonic	*MAOA*	Nonsynonymous SNV	0
chr12	52099402	52099402	G	T	Exonic	*SCN8A*	Nonsynonymous SNV	0
chr7	151000000	150695486	C	A	Exonic	*NOS3*	Nonsynonymous SNV	0
chr17	28544202	28544202	G	A	Exonic	*SLC6A4*	Synonymous SNV	0
chr13	47409054	47409054	A	G	Exonic	*HTR2A*	Nonsynonymous SNV	0
chr11	113000000	113295380	G	T	UTR5	*DRD2*		0
chr2	208000000	208432237	A	G	Exonic	*CREB1*	Synonymous SNV	0
chrX	114000000	114141765	G	A	Exonic	*HTR2C*	Synonymous SNV	0.000008
chr7	151000000	150696111	T	G	Exonic	*NOS3*	Nonsynonymous SNV	0.7530
chr22	19950040	19950040	C	T	Intronic	*COMT*		0.0001
chr17	1264520	1264520	G	A	Exonic	*YWHAE*	Synonymous SNV	0.0002
chr17	61574607	61574607	C	T	Exonic	*ACE*	Synonymous SNV	0.0033
chr11	637362	637373	GCGGGGGCATCT	–	Exonic	*DRD4*	Nonframeshift deletion of the *DRD4* gene	0.1452
chr17	61559923	61559923	C	T	Exonic	*ACE*	Synonymous SNV	0.4492
chr17	61564052	61564052	A	G	Exonic	*ACE*	Synonymous SNV	0.4999
chr17	61573761	61573761	T	C	Exonic	*ACE*	Synonymous SNV	0.5233
chr17	61574492	61574492	G	A	Intronic	*ACE*		0.5562
chr11	640119	640119	C	T	Exonic	*DRD4*	Synonymous SNV	0.9162
chr11	113000000	113283459	G	A	Exonic	*DRD2*	Synonymous SNV	0.4158

Three variants with a frequency of less than 0.5% in the general population were identified in more than one subject in our series, with two of these being synonymous variants. The first (NM_006761: c.444G>A; p.S148S) is in exon 4 of the *YWHAE* gene, identified in two samples as heterozygous (samples 100 and 57) with a frequency in ExAC_ALL of 2 in 1000 (0.0002) (rs761324586) and reported in the LOVD database as “likely benign” (Genomic variant #0000320090). The second is in the *ACE* gene (NM_001178057: exon13: c.1956C > T; p.I652I) and was identified in five casuistry samples (106, 82, 38, 64, 32) with heterozygosity and a frequency in the ExAC_ALL database of 0.0033 (rs144242912). This variant was reported with uncertain clinical significance in the ClinVar database (ID: 324420) in a case of renal dysplasia. The third variant was observed as heterozygous in two samples (24 and 64) and is a 10 bp intronic variant in the 5′ portion of exon 1 in the *COMT* gene (rs199755426). It was reported in the LOVD database as “likely benign”.

The synonymous variant c.870G>A, p.P290P in exon 6 of the *DRD2* gene (rs6277) is especially enriched in frequency in our series compared with the general population. In fact, its allelic frequency passes from 41% in ExAC_ALL to 55% in our series (corrected *P*-value = 0.0009463032). The synonymous variant c.1164G > A, p.K388K in exon 6 of *HTR2C* was observed in one male subject. This variant is extremely rare in the general population (<0.0008%).

From the results obtained, it is highlighted several potential genetic links with suicide in a cohort of Italians. This supports a relationship between specific genetic variants and suicidal behaviour in this population.

## Discussion

In our Tuscan cohort, males are 3–4 times more likely to take their own lives than females. This proportion is slightly higher than the ISTAT data, where male subjects were three times more frequent than females [3]. Independent of age, our results partially differ from national data. Globally, suicide rates increase with age. There are about 1.4 suicides per 100 000 inhabitants in persons under the age of 25, 6.1 per 100 000 in those between 25 and 44 years of age, 8.4 per 100 000 in those between 45 and 65 years of age, and up to 11.3 per 100 000 in those over 65 [3]. Our results regarding suicide modalities are consistent with the national data, in which the three main suicide modalities are: hanging and suffocation (48.9%), jumping (19.2%), and use of firearms (11.3%) [3]. Our findings for suicide motives are also consistent with the nationwide findings. Our research and the national data both suggest the main cause to be psychiatric disorders [3].

Here, we have investigated genetic variants in genes important for neurobiological pathways linked to suicidal behaviour among patients with co-existing psychiatric illnesses. In this study, we have analysed genes that are intimately related to numerous neurobiological pathways considered to be important for the pathogenesis of suicide and/or associated disorders.

Examination of genetic data allowed us to identify 19 variants in our series with different frequencies that are statistically more significant than the reference population frequencies. In our subjects, seven variants were not observed, seven had a lower frequency, and three had a frequency of less than 0.5%. Notably, seven of these polymorphisms had a lower frequency in our series than in the general population, possibly suggesting a protective effect. These variants are in *ACE*, *NOS3*, and *DRD4*. In particular, the latter gene suggests the need for further investigation of *DRD4* haplotypes and suicide rates, especially in males. Because *DRD4* is located in a CpG island, investigating any sex-specific differences in methylation status at this locus may help illuminate a mechanism underlying the haplotypic association observed in our study. *DRD4* does not appear to be related to the VNTR in exon 3. In fact, *DRD4* association studies have primarily focused on this VNTR, a 48-bp repeat polymorphism in the third exon that results in varying lengths of the third intracellular loop in the D4 receptor [17, 19]. The most commonly occurring repeat length variants are the 2-(2 R), 4-(4 R), and 7-(7 R) repeats. Other rare repeat length variants, however, are also observed [19]. Although a synonymous SNP would not influence the protein sequence, it may have an impact on mRNA splicing. Abnormalities in dopaminergic activity in the nigrostriatal system have been suggested to be most often related to schizophrenia patients. The rs762502 variant especially changes from a frequency of 91% in the general population to 60% in our series (116 alleles/196), and has previously been reported in the literature as linked to tardive diskynesia [19].

The *DRD2* gene 957 C > T polymorphism is associated with post-traumatic stress disorder in war veterans. In a study of 300+ Russian patients with schizophrenia, the rs6277(C) allele was associated with higher risk [42]. From a pooled meta-study, the allelic odds ratio was 1.42 (CI: 1.26–1.61, *P* < 0.00005) and the genotypic odds ratio for the (C; C) genotype was 1.6 (CI: 1.32–1.95, *P* < 0.00005) [43]. A significant association (*P* = 0.021) was found between the C allele and post-traumatic stress disorder in a sample of 127 (assumedly Australian) war veterans with 228 controls. The T allele has been associated with better learning from positive and negative outcomes in a young adult sample [19] while the CC genotype has been associated with poorer overall cognitive ability in an older Scottish population sample [44], possibly suggesting the learning those with T alleles may better learn into older age. Biologically, T alleles at rs6277 have been associated with a decreased affinity of D2 receptors in the striatum [44].

The relevance of the seven unique variants in the general population is possibly significant in the individual suicide cases we examined. Specifically, the missense variants of the *MAOA*, *HTR2A*, *SCN8A*, and *NOS3* genes produce a change in the protein products, potentially altering the biophysical properties and functions of these proteins. We used three different predictive tools to evaluate the clinical significance of these variants. Of the four potentially harmful variants, only one (*HTR2A*) was categorised as tolerated/benign according to two prediction tools. The SNPs in *SCN8A* and *NOS3* were potentially causative of damage per the MutationTaster tool, while the NM_001270458 mutation in the *MAOA* gene was labelled as potentially deleterious/damaging by two prediction tools.

Deeper insight into clinical and biological risk factors will help us more fully understand this model of suicidal behaviour and establish new therapies and tailored interventions. The molecular pathology of suicidal behaviour remains complex; epidemiological studies have demonstrated familial clustering of suicide and suicidal behaviour [24, 38]. The causative role of SNPs in suicide predisposition must be confirmed, and a deeper understanding of the molecular mechanisms is critical to ensure the biological significance of these data.

An increased sample size of both cases of individuals who have attempted suicide and those who have committed suicide could improve the accuracy of our examinations. DNA extracted from suicide cases can be used to investigate VNTRs in specific genes, such as *MAOA* or *COMT*. Furthermore, though the mechanism and magnitude of the genetic contribution in the emergence of suicidal behaviour are still poorly understood, this work could be included in future meta-analyses on larger samples.

Familial, adoption, and twin studies suggest a heritability component to suicidal behaviour. Few data have been previously reported on Italian suicidal behaviour based on genetic studies. Here, 19 gene variants were identified with frequencies that were statistically different from those of the general population. Of these, seven variants have never been observed, seven had a lower frequency, and three variants had a lower than 0.5% frequency and were observed in more than one subject, suggesting a possible role in suicidal behaviour.

It is now well-established that genetic variants in numerous neurobiological pathways are potentially relevant in the pathogenesis of suicide. Considering the limitations and low sample number of our study, however, we are cautious in proposing a role for the specific genes examined here in the manifestation of suicidal behaviour. Nevertheless, consistent with numerous lines of evidence demonstrating that suicide is polygenic, we believe our work is relevant and provides important insight into the relationship between genes and suicide risk in the Italian population.
